# Analysis of Risk Prediction Model for Recurrence of Trigeminal Neuralgia After Percutaneous Balloon Compression

**DOI:** 10.1155/prm/6688829

**Published:** 2026-01-07

**Authors:** Ying Guo, Jing Feng, Yige Ma, Na Zhang, Jianheng Gu, Zhaoting Pei

**Affiliations:** ^1^ Harbin Medical University, Harbin, 150081, China, hrbmu.edu.cn; ^2^ Department of Central Catheter Room, The Fourth Affiliated Hospital of Harbin Medical University, Harbin, 150001, China, hrbmu.edu.cn; ^3^ Department of Neurosurgery, The Fourth Affiliated Hospital of Harbin Medical University, Harbin, 150001, China, hrbmu.edu.cn

**Keywords:** machine learning, percutaneous balloon compression, predictive model, recurrence, trigeminal neuralgia

## Abstract

**Objective:**

Trigeminal neuralgia (TN) is a debilitating disorder characterized by severe facial pain. While percutaneous balloon compression (PBC) is an effective surgical treatment for TN, recurrence remains a significant concern, with varying reported rates. The identification of factors that contribute to recurrence after PBC is critical for improving treatment outcomes. However, existing predictive models for recurrence have limitations in accuracy and generalizability. This study aims to explore the influencing factors of TN recurrence after PBC and to construct a TN recurrence risk prediction model.

**Methods:**

The clinical data of 448 TN patients treated for PBC were retrospectively analyzed and divided into a modeling group (*n* = 317) and a validation group (*n* = 131) in a ratio of 7:3. Patients were divided into two groups based on whether they experienced recurrence or not. Risk prediction models were constructed using three machine learning methods: logistic regression, random forest, and XGBoost. The area under the receiver operating characteristic curve (AUC), accuracy, sensitivity, and specificity were used to evaluate the model performance.

**Results:**

Multivariate analysis showed that the duration of disease, pain type, balloon shape, compression time, and delayed disappearance of pain were influencing factors for TN recurrence after PBC, while facial numbness was a protective factor. All three predictive models exhibit high accuracy. In the modeling group, the AUC values for the logistic regression, random forest, and XGBoost models are 0.810, 0.824, and 0.816, respectively. Furthermore, the random forest model outperforms the other two models in terms of accuracy, sensitivity, and specificity. Additionally, external validation also demonstrates that the random forest model has good predictive value for TN after PBC (AUC = 0.835).

**Conclusion:**

The random forest model showed excellent performance in predicting TN recurrence after PBC, providing a powerful reference for clinical prevention.

## 1. Introduction

Trigeminal neuralgia (TN) is a common craniocerebral neuropathy characterized by recurrent, paroxysmal, electric shock‐like, and tearing severe pain in the facial area innervated by the trigeminal nerve [1]. In recent years, percutaneous balloon compression (PBC) has gradually become one of the commonly used surgical treatments for TN, although microvascular decompression is still considered the primary treatment for most classical TN cases [2, 3]. However, PBC is associated with a recurrence rate of 20%–43% [4–6]. Recurrence in TN patients complicates their condition, subjecting them to repeated pain episodes and prolonged treatment, which can cause significant physiological and psychological distress, exacerbate the treatment burden, and, in severe cases, lead to anxiety and depression [7, 8]. Risk prediction models have the potential to identify high‐risk patients early and facilitate timely interventions, which may reduce the likelihood of PBC recurrence and alleviate the treatment burden. Although research on PBC recurrence risk prediction models has commenced, limitations such as model simplicity, insufficient efficacy, and lack of external validation have hindered their universal applicability and reliability [9, 10]. Therefore, this study collected clinical data of patients with TN after PBC surgery and constructed three risk prediction models, namely, logistic regression, random forest, and XGBoost, in order to improve prediction accuracy, achieve accurate evaluation, and provide a scientific basis for clinical decision‐making. The risk prediction model can identify high‐risk patients in advance, enabling clinicians to make informed decisions about the timing of PBC, prioritize patients for early intervention, or modify perioperative management strategies, which may reduce the likelihood of PBC postoperative recurrence and alleviate the treatment burden. External validation was used to ensure universality and promote clinical management and research progress of pain recurrence after PBC surgery.

## 2. Materials and Methods

### 2.1. Study Subjects

This study was a retrospective study, and TN patients who underwent PBC treatment in the Fourth Affiliated Hospital of Harbin Medical University and the First Affiliated Hospital of Harbin Medical University from January 2020 to December 2023 were selected as research subjects. Patients from the Fourth Affiliated Hospital of Harbin Medical University were assigned to the modeling group (*n* = 317), while patients from the First Affiliated Hospital of Harbin Medical University were assigned to the validation group (*n* = 131). Inclusion criteria included the following: (1) patients meeting the diagnostic criteria for TN as specified in the third edition of the International Classification of Headache Disorders (ICHD‐3) [11]; (2) patients aged ≥ 18 years; and (3) patients with TN postpercutaneous microballoon compression surgery. Exclusion criteria included the following: (1) patients with incomplete clinical data and (2) patients with a history of cognitive impairment or psychiatric disorders.

This study utilized the Events Per Variable (EPV) formula for calculating the sample size required for clinical prediction models, which recommends having 10 positive events for each predictor variable [12, 13]. The formula is *N* = 10×(*K*/*P*), where *P* represents the minimum proportion of negative or positive cases in the population and *K* is the number of independent variables. Combined with the results of previous studies, the recurrence rate of pain after PBC surgery is 25.4% and there are five to six independent influencing factors after PBC surgery. Therefore, in this study, *P* = 0.254, *K* = 10, and considering the 10% loss rate, the total sample size was finally calculated to be 437 cases. Considering the 70% and 30% allocation principle for the modeling group and the verification group [14], the sample size of the modeling group needs to be at least 306 cases, and the sample size of the verification group needs to be at least 131 cases.

### 2.2. Research Methods

#### 2.2.1. Criteria for Recurrence After PBC

The Barrow Neurological Institute Pain Intensity Score (BNI‐P) was used to assess the degree of postoperative pain relief. Grade I: complete pain relief without any medication; Grade II: occasional pain that does not require medication; Grade III: pain that is sometimes present but can be fully controlled with medication; Grade IV: pain that persists and cannot be fully controlled with medication; and Grade V: persistent pain without relief. “Recurrence” of pain was defined as an increase in pain severity to BNI Grades III–V or the need for reoperation after initially achieving Grade I pain relief. Higher BNI grades reflect more severe pain and less effective pain control [15]. The efficacy of PBC was assessed using the BNI‐P. Pain relief was considered complete if patients achieved BNI Grade I, meaning no pain without the need for medication, immediately after the procedure. Patients who reported pain on Day 3 postsurgery were not classified as having recurrence, as this pain was considered part of the early recovery phase rather than true recurrence. The follow‐up was primarily conducted via telephone, combined with outpatient and inpatient medical records of patients who revisited the hospital, to document cases of postoperative recurrence. Patients who did not experience recurrence were telephonically inquired about whether their BNI Grade reached III–V or whether they underwent reoperation during the period from the postoperative date to April 20, 2024, with PBC recurrence serving as the outcome event.

#### 2.2.2. Survey Tool

After systematically reviewing domestic and foreign literature and referring to expert opinions, we designed the “Survey of Factors Affecting TN Recurrence after PBC Surgery,” which ultimately included 3 parts and 23 indicators. (1) Patient individual factors: gender, age, smoking history, drinking history, duration of disease, side, branch, pain type, previous surgery history, acupuncture history, closed treatment history, drug treatment history, drug treatment effect, hypertension, diabetes, hyperlipidemia, and history of cerebrovascular disease; (2) intraoperative factors: balloon shape, compression time, balloon volume, and intraoperative bleeding; and (3) postoperative factors: delayed disappearance of pain and facial numbness.

#### 2.2.3. Surgical Technique and Methodology

The PBC procedure was performed under general anesthesia with the patient in a supine position and the head extended. The foramen ovale was cannulated under fluoroscopic guidance in both anteroposterior and lateral views to ensure accurate needle placement. The “pear‐shaped” balloon contour was confirmed under fluoroscopy prior to compression. A balloon was inflated with contrast agent to achieve compression of the Gasserian ganglion for the treatment of TN. The procedure was conducted according to the standard protocols at the institution.

#### 2.2.4. Data Collection and Quality Control

A research group on the topic of postoperative recurrence of PBC in TN patients was established. It included one director of the pain department, who was responsible for guiding the questionnaire content; one head nurse, who was responsible for grasping the progress of the project; and three nursing graduate students, who were responsible for literature retrieval, questionnaire preparation, data collection, and data analysis. The self‐designed “Questionnaire on Factors Affecting TN Recurrence after PBC Surgery” was used to complete data collection through the hospital electronic medical record system and nursing record sheets. Before the formal data collection, the three nursing graduate students were trained in the operation of the electronic medical record system, data collection, and entry; the data collection process strictly followed the double‐check system, in which two nursing graduate students were responsible for checking the consistency of the collected information with the original information, and the other nursing graduate student was responsible for keeping the data and checking 10% of the data to ensure that the input information was accurate; data with missing data greater than 15% were not included in the study.

### 2.3. Statistical Methods

SPSS 27.0 and R 4.3.3 software were used for data collation and analysis. Enumeration data were described by number of cases and percentage (%), and the differences between groups were compared by the chi‐squared test and Fisher’s exact probability method. Variables with statistically significant differences (*p*  <  0.05) in univariate analysis were included in multivariate logistic regression analysis to screen the influencing factors of postoperative recurrence of PBC. The risk prediction model was constructed using the “rms,” “random forest,” and “XGBoost” packages in R software, and the robustness of the model was further improved by cross‐validation. The model’s performance was evaluated using AUC, accuracy, sensitivity, and specificity. AUC measures the model’s ability to distinguish between positive and negative outcomes, with values closer to 1 indicating better performance. Accuracy is the proportion of correct classifications (true positives + true negatives)/total cases. Sensitivity (recall) is the proportion of true positives correctly identified, while specificity is the proportion of true negatives identified. An AUC above 0.7 and accuracy, sensitivity, and specificity values above 70% were considered indicative of good model performance.

## 3. Results

### 3.1. Characteristics of the Enrolled Study Subjects

A total of 463 clinical records of TN patients who underwent PBC were initially collected. This cohort included 326 patients from the Fourth Affiliated Hospital of Harbin Medical University and 137 patients from the First Affiliated Hospital of Harbin Medical University. Fifteen patients were subsequently excluded due to reasons such as missing clinical data or inability to be contacted. Consequently, the final study population comprised 448 patients. Among them, 317 patients from the Fourth Affiliated Hospital were assigned to the model development group, and 131 patients from the First Affiliated Hospital were assigned to the validation group.

The final cohort of 448 TN patients treated with PBC consisted of 163 males (36.4%) and 285 females (63.6%). Regarding age distribution, 159 patients (35.5%) were aged ≤ 65 years, 157 patients (35.0%) were aged 66 to < 75 years, and 132 patients (29.5%) were aged ≥ 75 years. Pain recurrence after PBC was observed in 137 patients, corresponding to a recurrence rate of 30.4%. Comparative analysis of baseline characteristics revealed no statistically significant differences between the model development and validation groups (*p*  >  0.05). This balance mitigates potential bias in the study results that could arise from an uneven distribution of variables **(**Table 1 **).**

**Table 1:** Comparison of baseline characteristics between the model development and validation groups.

Characteristic	Category	Model development group (*n* = 317)	Validation group (*n* = 131)	*p*
Demographics				
Age	≤ 65 years	114 (36.0%)	45 (34.4%)	0.535
	66–75 years	113 (35.6%)	44 (33.6%)	
	≥ 75 years	90 (28.4%)	42 (32.1%)	
Sex	Male	115 (36.3%)	48 (36.6%)	0.924
	Female	202 (63.7%)	83 (63.4%)	
Personal history and disease duration				
Smoking history	Yes	60 (18.9%)	32 (24.4%)	0.19
	No	257 (81.1%)	99 (75.6%)	
Alcohol history	Yes	53 (16.7%)	29 (22.1%)	0.177
	No	264 (83.3%)	102 (77.9%)	
Disease duration	< 5 years	180 (56.8%)	67 (51.1%)	0.545
	5–10 years	66 (20.8%)	38 (20.9%)	
	> 10 years	71 (22.4%)	26 (19.8%)	
Disease characteristics				
Involved branch(es)	V1	19 (6.0%)	5 (3.8%)	0.428
	V2	78 (24.6%)	35 (26.9%)	
	V3	74 (23.3%)	29 (22.3%)	
	V1+V2	39 (12.3%)	20 (15.4%)	
	V1+V3	8 (2.5%)	3 (2.3%)	
	V2+V3	66 (20.8%)	32 (24.6%)	
	V1+V2+V3	33 (10.4%)	6 (4.6%)	
Affected side	Left	137 (43.2%)	52 (39.7%)	0.492
	Right	180 (56.8%)	79 (60.3%)	
Pain type	Typical TN	294 (92.7%)	118 (90.1%)	0.345
	Atypical TN	23 (7.3%)	13 (9.9%)	
Previous treatment history				
Previous surgery	None	259 (81.7%)	107 (81.7%)	0.591
	Microvascular decompression	32 (10.1%)	16 (12.2%)	
	Radiofrequency thermocoagulation	21 (6.6%)	5 (3.8%)	
	Gamma knife	5 (1.6%)	3 (2.3%)	
Acupuncture history	Yes	67 (21.1%)	23 (17.6%)	0.39
	No	250 (78.9%)	108 (82.4%)	
Nerve block history	Yes	17 (5.4%)	11 (8.4%)	0.228
	No	300 (94.6%)	120 (91.6%)	
Previous medication	Carbamazepine	266 (83.9%)	106 (80.9%)	0.525
	Oxcarbazepine	27 (8.5%)	16 (12.2%)	
	Mecobalamin	12 (3.8%)	3 (2.3%)	
	Other	12 (3.8%)	6 (4.6%)	
Reason for PBC	Medication‐resistant	177 (55.8%)	72 (55.0%)	0.866
	Intolerable side effects	140 (44.2%)	59 (45.0%)	
Comorbidities				
Hypertension	Yes	148 (46.7%)	58 (44.3%)	0.641
	No	169 (53.3%)	73 (55.7%)	
Diabetes mellitus	Yes	78 (24.6%)	22 (16.8%)	0.071
	No	239 (75.4%)	109 (83.2%)	
Hyperlipidemia	Yes	27 (8.5%)	11 (8.4%)	0.967
	No	290 (91.5%)	120 (91.6%)	
Cerebrovascular disease	Yes	136 (42.9%)	53 (40.5%)	0.634
	No	181 (57.1%)	78 (59.5%)	
Procedure‐related factors				
Balloon shape	Pear‐shaped	288 (90.9%)	112 (85.5%)	0.095
	Nonpear‐shaped	29 (9.1%)	19 (14.5%)	
Compression time	≤ 120s	228 (71.9%)	83 (63.4%)	0.073
	> 120 s	89 (28.1%)	48 (36.6%)	
Balloon volume	< 0.6 mL	78 (24.6%)	27 (20.6%)	0.172
	0.6–0.7 mL	98 (30.9%)	33 (25.2%)	
	> 0.7 mL	141 (44.5%)	71 (54.2%)	
Intraoperative bleeding	Yes	33 (10.4%)	20 (15.3%)	0.148
	No	284 (89.6%)	111 (84.7%)	
Postoperative outcomes				
Delayed disappearance of pain	Yes	42 (13.2%)	23 (17.6%)	0.239
	No	275 (86.8%)	108 (82.4%)	
Facial numbness	Yes	253 (79.8%)	94 (71.8%)	0.063
	No	64 (20.2%)	37 (28.2%)	

### 3.2. Univariate Analysis Results of General Information and Factors Influencing Recurrence in the Modeling Group

A total of 317 patients were included in the modeling group, including 115 males (36.3%) and 202 females (63.7%); 114 patients (36.0%) were aged ≤ 65 years, 113 (35.6%) were aged 66–74 years, and 90 (28.4%) were aged ≥ 75 years. The incidence of recurrence after PBC was 34.1%. Based on whether the patients experienced recurrence, the modeling group was divided into a recurrence group (*n* = 108) and a nonrecurrence group (*n* = 209). Immediately following the PBC procedure, all patients who underwent the procedure achieved pain relief, with the majority reaching BNI Grade I (no pain without medication). Univariate analysis was conducted on the general information of patients in the two groups. The results showed that there were statistically significant differences between the two groups in terms of duration of illness, TN pain type, history of previous surgery, history of hypertension, history of diabetes, balloon shape, compression duration, balloon volume, delayed disappearance of pain, and facial numbness (*p*  <  0.05). The details are given in Table 2.

**Table 2:** General data of the subjects in the modeling group and results of univariate analysis of relapse influencing factors.

Project	Number of cases	Recurrence (*n* = 108)	Group without recurrence (*n* = 209)	*p*
*Age (years)*				0.442
≤ 65	114	36 (31.6%)	78 (68.4%)	
66–74	113	39 (34.5%)	74 (65.5%)	
≥ 75	90	33 (36.7%)	57 (63.3%)	

*Sex*				0.303
Male	115	35 (30.4%)	80 (69.6%)	
Female	202	73 (36.1%)	129 (63.9%)	

*Smoking history*				0.298
Yes	60	17 (28.3%)	43 (71.7%)	
No	257	91 (35.4%)	166 (64.6%)	

*Drinking history*				0.332
Yes	53	15 (28.3%)	38 (71.7%)	
No	264	93 (35.2%)	171 (64.8%)	

*Duration of disease (years)*				< 0.001
< 5	180	34 (18.9%)	146 (81.1%)	
5–10	66	34 (51.5%)	32 (48.5%)	
> 10	71	40 (56.3%)	31 (43.7%)	

*Side*				0.426
Left	137	50 (36.5%)	87 (63.5%)	
Right	180	58 (32.2%)	122 (67.8%)	

*Branch*				0.63
V1	19	7 (36.8%)	12 (63.2%)	
V2	78	23 (29.5%)	55 (70.5%)	
V3	74	24 (32.4%)	50 (67.6%)	
V1+2	39	17 (43.6%)	22 (56.4%)	
V1+3	8	4 (50.0%)	4 (50.0%)	
V2+3	66	20 (30.3%)	46 (69.7%)	
V1+2 + 3	33	13 (39.4%)	20 (60.6%)	

*Pain type*				< 0.001
Typical	294	92 (31.3%)	202 (68.7%)	
Atypical	23	16 (69.6%)	7 (30.4%)	

*Previous surgical history*				0.003
No	259	77 (29.7%)	182 (70.3%)	
Microvascular decompression	32	19 (59.4%)	13 (40.6%)	
Radiofrequency thermocoagulation	21	10 (47.6%)	11 (52.4%)	
Gamma knife	5	2 (40.0%)	3 (60.0%)	

*Acupuncture history*				0.81
Yes	67	22 (32.8%)	45 (67.2%)	
No	250	86 (34.4%)	164 (65.6%)	

*Block therapy history*				0.677
Yes	17	5 (29.4%)	12 (70.6%)	
No	300	103 (34.3%)	197 (65.7%)	

*Drug treatment history*				0.24
Carbamazepine	266	92 (34.6%)	174 (65.4%)	
Oxcarbazepine	27	11 (40.7%)	16 (59.3%)	
Mecobalamin	12	1 (8.3%)	11 (91.7%)	
Other	12	4 (33.3%)	8 (66.7%)	

*Drug therapeutic effect*				0.756
No response	177	59 (33.3%)	118 (66.7%)	
Side effects of medication	140	49 (35.0%)	91 (65.0%)	

*Hypertension history*				0.012
Yes	148	61 (41.2%)	87 (58.8%)	
No	169	47 (27.8%)	122 (72.2%)	

*Diabetes history*				0.041
Yes	78	34 (43.6%)	44 (56.4%)	
No	239	74 (31.0%)	165 (69.0%)	
*Hyperlipidemia*				0.075
Yes	27	5 (18.5%)	22 (81.5%)	
No	290	103 (35.5%)	187 (64.5%)	

*Cerebrovascular disease history*				0.873
Yes	136	47 (34.6%)	89 (65.4%)	
No	181	61 (33.7%)	120 (66.3%)	

*Balloon shape*				< 0.001
Pear‐shaped	288	87 (30.2%)	201 (69.8%)	
Nonpear‐shaped	29	21 (72.4%)	8 (27.6%)	

*Compression time (s)*				0.009
≤ 120	226	67 (29.6%)	159 (70.4%)	
> 120	91	41 (45.0%)	50 (55.0%)	

*Intraoperative bleeding*				0.63
Yes	33	10 (30.3%)	23 (69.7%)	
No	284	98 (34.5%)	186 (65.5%)	

*Balloon volume (mL)*				0.048
< 0.6	78	35 (44.9%)	43 (55.1%)	
0.6–0.7	98	27 (27.6%)	71 (72.4%)	
> 0.7	141	46 (32.6%)	95 (67.4%)	

*Delayed disappearance of pain*				< 0.001
Yes	42	24 (57.1%)	18 (42.9%)	
No	275	84 (30.5%)	191 (69.5%)	

*Facial numbness*				< 0.001
Yes	253	73 (28.9%)	180 (71.1%)	
No	64	35 (54.7%)	29 (45.3%)	

### 3.3. Results of Logistic Regression Analysis on Factors Influencing Recurrence of TN After PBC

Ten variables with statistically significant differences in the univariate analysis were selected as independent variables for logistic regression analysis, with TN recurrence as the dependent variable. The multivariate analysis revealed that disease duration > 5 years, atypical pain type, nonpear‐shaped balloon, compression duration > 120s, delayed disappearance of pain, hypertension history, diabetes history, balloon volume, and facial numbness as a protective factor were significantly associated with TN recurrence after PBC (*p*  <  0.05). In contrast, history of hypertension, history of diabetes, and balloon volume also showed significant associations with TN recurrence (*p*  <  0.05) but were excluded from the final model due to factors such as collinearity with other variables and lower clinical relevance. The details are given in Table 3.

**Table 3:** Results of logistic regression analysis of influencing factors for postoperative recurrence in the modeling group.

Variable	Category	Regression coefficient (B)	Standard error (SE)	Wald *χ* ^2^	*p*	OR (95% CI)
Constant		−1.543	0.497	9.638	0.002	
Disease duration	< 5 Years (Ref)			28.186	< 0.001	
	5–10 Years	1.216	0.38	10.248	0.001	3.37 (1.60–7.11)
	> 10 Years	1.807	0.354	26.106	< 0.001	6.10 (3.05–12.19)
Pain type	Typical (Ref)					
	Atypical	1.838	0.577	10.156	0.001	6.29 (2.03–19.47)
Balloon shape	Pear‐shaped (Ref)					
	Nonpear‐shaped	1.865	0.528	12.486	< 0.001	6.46 (2.30–18.17)
Compression time	≤ 120 s (Ref)					
	> 120 s	0.85	0.334	6.461	0.011	2.34 (1.22–4.51)
Delayed disappearance of pain	No (Ref)					
	Yes	1.311	0.439	8.931	0.003	3.71 (1.57–8.76)
Facial numbness	No (Ref)					
	Yes	−1.242	0.358	12.052	< 0.001	0.29 (0.14–0.58)
Balloon volume	< 0.6 mL (Ref)			7.543	0.023	
	0.6–0.7 mL	0.75	0.345	4.728	0.03	0.75 (0.45–1.25)
	> 0.7 mL	0.65	0.317	4.207	0.04	0.65 (0.40–1.05)
Hypertension history		1.082	0.432	6.271	0.012	2.95 (1.26–6.89)
Diabetes history		1.099	0.539	4.158	0.041	3.00 (1.04–8.63)
Previous surgical history	No surgery (Ref)			2.451	0.484	
	Microvascular decompression	0.5	0.45	1.235	0.266	1.65 (0.68–3.99)
	Radiofrequency thermocoagulation	0.38	0.42	0.818	0.366	1.46 (0.64–3.32)
	Gamma knife	0.62	0.61	1.033	0.309	1.86 (0.56–6.15)

Although 10 variables showed significant associations (*p*  <  0.05) in the univariate analysis, six variables were selected for the final model due to their strong statistical significance (with *p* values well below 0.001) and clinical relevance in predicting TN recurrence. These six variables exhibited robust associations with TN recurrence and are clinically relevant, with their inclusion in the model enhancing its predictive power.

### 3.4. Construction and Validation of a Risk Prediction Model for TN Recurrence After PBC Surgery

**Figure 1:**
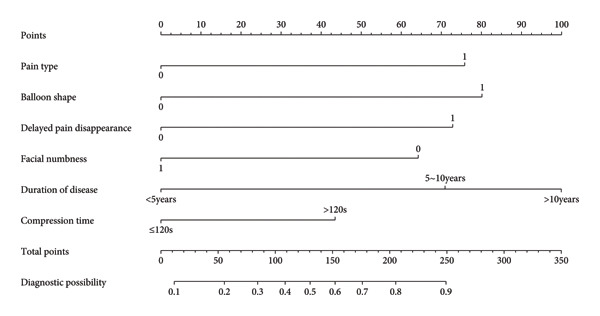
Nomogram for risk of recurrence after PBC surgery.

According to the six predictive factors screened by the results of logistic regression analysis, three machine learning algorithms, logistic regression, random forest, and XGBoost, were used to build a prediction model for TN recurrence after PBC surgery. The results of the logistic regression model were visualized, and a nomogram for TN recurrence after PBC surgery was drawn. A vertical line was drawn from each factor to the “points” to obtain the corresponding score. The total score of the nomogram was obtained by adding up the scores of all factors. The probability corresponding to the total score was the risk value of TN recurrence after PBC surgery. For example, if the patient’s disease duration is > 10 years, the TN type is pain type, the balloon shape is not pear‐shaped, the compression time is > 120 s, there is no delayed disappearance of pain after surgery, and facial numbness occurs after surgery, the patient’s total score is 100 + 0+70 + 40+0 + 0 = 210 points, and the probability of pain recurrence is about 80%. The nomogram is shown in Figure 1 and Table 4. After fivefold cross‐validation, the AUC values of the three models in the modeling group ranged from 0.810 to 0.824, among which the AUC value of the random forest model was 0.824, the accuracy was 0.793, the sensitivity was 0.801, and the specificity was 0.759, all higher than other models. The optimal random forest model was verified based on the validation group, and its AUC value was 0.835, the accuracy was 0.803, the sensitivity was 0.813, and the specificity was 0.772, indicating that the model has good prediction performance. The prediction performance and ROC curves of each model are shown in Table 5 and Figure 2.

**Table 4:** Point assignments and risk stratification for the nomogram predicting pain recurrence after PBC.

Variable	Category	Assigned points
Pain type	Typical (Ref)	0
	Atypical	100
Balloon shape	Pear‐shaped (Ref)	0
	Nonpear‐shaped	100
Delayed disappearance of pain	No (Ref)	0
	Yes	70
Facial numbness	No (Ref)	0
	Yes	−65
Duration of disease	< 5 Years (Ref)	0
	5–10 Years	65
	> 10 Years	95
Compression time	≤ 120s (Ref)	0
	> 120s	45

**Risk category**	**Total points range**	**Estimated probability of recurrence**

Low risk	< 100	< 20%
Intermediate‐low risk	100–150	20%–30%
Intermediate‐high risk	150–250	30%–70%
High risk	> 250	> 70%

*Note:* Instructions for use: For each patient, determine the points for all variables. Sum all points to calculate the total points. Identify the risk category and the corresponding estimated probability from the table above.

**Table 5:** Evaluation results of three kinds of prediction models for trigeminal neuralgia recurrence after percutaneous microballoon compression.

Prediction model	AUC	Accuracy	Sensitivity	Specificity
Modeling group for logistic regression model	0.810	0.758	0.761	0.736
Validation group for logistic regression model	0.817	0.779	0.793	0.750
Modeling group for random forest model	0.824	0.793	0.801	0.759
Validation group for random forest model	0.835	0.803	0.813	0.772
Modeling group for XGBoost model	0.816	0.792	0.798	0.757
Validation group for XGBoost model	0.822	0.797	0.809	0.769

Figure 2Receiver operating characteristic (ROC) curves: (a) modeling group and (b) validation group.

**Figure:**
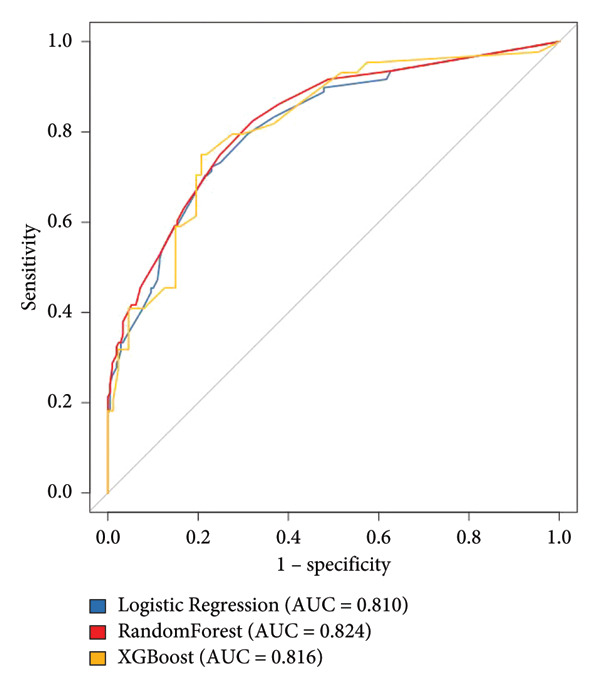
(a)

**Figure:**
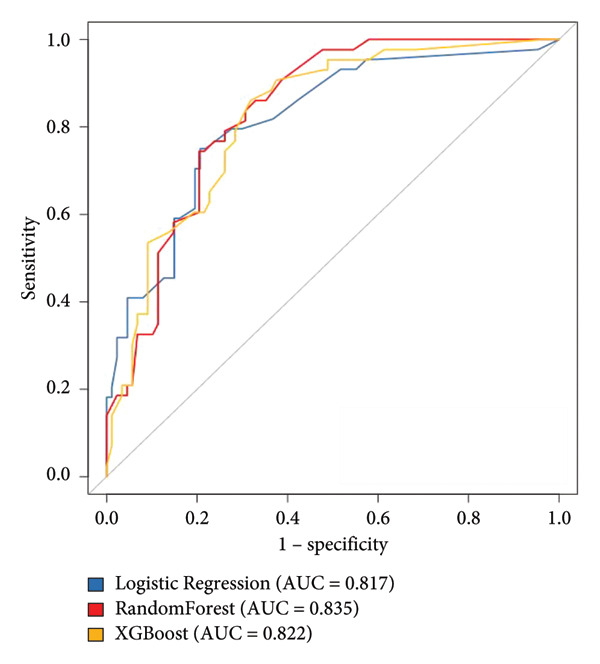
(b)

## 4. Discussion

PBC treatment of TN is widely used in clinical practice and has gradually become a mainstream treatment method due to its minimally invasive and high safety characteristics [16]. PBC for TN patients can effectively reduce pain and improve their quality of life, but there is a generally high recurrence rate after surgery. In this study, 317 TN patients were included in the modeling group, with a pain recurrence rate of 34.1%, indicating a still high recurrence risk. Foreign studies have shown that the recurrence rate of TN pain after PBC is 20%–43%; domestic studies have reported recurrence rates of 25.4%–39.5% after PBC [17, 18]. The significant variation in pain recurrence rates after PBC among different studies may be related to factors such as pain recurrence assessment tools, follow‐up duration, and postoperative management. Therefore, establishing an accurate risk prediction model for TN recurrence after PBC is of great significance for early identification of high‐risk patients after surgery, in order to improve treatment plans and prognosis.

The results of this study indicate that the risk of TN recurrence after PBC significantly increases with disease duration, with patients having a disease duration of 5–10 years and over 10 years being more prone to recurrence compared to those with a duration of less than 5 years. This aligns with the findings of Ding [19]. Long‐term pain may lead to central nervous system sensitization, and even after PBC surgery compresses the Gasserian ganglion to alleviate pain associated with TN, patients’ pain perception systems remain highly sensitive, thus making them more susceptible to pain recurrence [20]. Atypical pain is also a factor influencing TN recurrence after PBC, consistent with the research results of Grewal et al. [21]. Patients with atypical pain often present with poorly defined or generalized trigger points, which complicates the identification of the primary pain source. This difficulty in precise targeting can lead to suboptimal surgical outcomes, increasing the likelihood of recurrence, as noted in previous studies [22, 23]. Notably, patients with atypical pain also have a higher risk of pain recurrence after microvascular decompression surgery [24], which draws a similar conclusion to this study. Therefore, healthcare professionals are advised to strengthen the promotion of health education on TN, aiming to detect symptoms early, intervene promptly for treatment, slow down disease progression, improve patients’ quality of life, and alleviate their pain. On the other hand, for patients with atypical pain, healthcare professionals should conduct detailed preoperative assessments to develop personalized and comprehensive treatment plans.

The results of this study indicate that a nonpear‐shaped balloon during surgery and a compression time exceeding 120 s are factors influencing TN recurrence after PBC. This aligns with the findings of Montano [25]. It is speculated that a nonpear‐shaped balloon may result in uneven pressure distribution near the Gasserian ganglion during compression [26]. This uneven distribution is unfavorable for blocking the pain transmission pathway, increasing the risk of nerve damage due to uneven compression and thus leading to a higher risk of pain recurrence for patients. In recent years, there has been controversy regarding the duration of balloon compression. The study indicates that a balloon compression duration of 60–120 s can effectively alleviate pain. Kourilsky et al. indicated in their study that a balloon compression time greater than 60 s is associated with TN recurrence after PBC [26], while the results of this study suggest that a compression time exceeding 120 s may be related to TN recurrence after PBC. This discrepancy may be due to inconsistent factors such as balloon volume, balloon pressure, and balloon shape in the studies, making it impossible to exclude the impact of these factors on prognosis. Therefore, healthcare professionals are advised to comprehensively analyze personalized data such as the patient’s age, pain duration, and pain intensity and select the most suitable balloon shape and compression time for each patient to maximize the surgical treatment effect, reduce postoperative complications, and decrease the recurrence rate after PBC.

The results of this study show that the delayed disappearance of postoperative pain is a factor influencing pain recurrence, consistent with the research of Lv [27], which reported a recurrence rate of up to 40.7% in patients with delayed disappearance of postoperative pain. The cause of delayed disappearance of postoperative pain may be related to high preoperative medication doses and a nonpear‐shaped balloon during surgery. It is speculated that the main reason for pain recurrence is the incomplete blockade of nerve conduction, with some nerve fibers potentially reconducting pain signals during recovery [28]. Additionally, the results of this study indicate that facial numbness after surgery is a protective factor against TN recurrence after PBC. The occurrence of facial numbness suggests that the trigeminal nerve has been sufficiently compressed and damaged, effectively blocking the conduction of pain signals and thus reducing the probability of pain recurrence. Facial numbness is often a sign of successful surgery and reflects the patient’s prognosis to some extent [29]. Therefore, healthcare professionals are advised to immediately assess the patient’s visual analog scale (VAS) score after surgery. Patients with delayed disappearance of postoperative pain can continue to take anticonvulsant medications as prescribed, with the dose appropriately reduced compared to preoperative levels. Personalized follow‐up plans should be developed for each patient’s specific situation to understand their postoperative recovery and provide health guidance.

Currently, the construction of TN recurrence risk prediction models after PBC in clinical practice mainly adopts traditional regression methods. In recent years, machine learning, as an important branch of artificial intelligence, has been widely applied in the healthcare field, demonstrating great potential in mining and processing medical data. It can not only complement the deficiencies of linear models but also play a crucial role in clinical decision support and personalized treatment [30]. This study takes the binary classification problem of whether TN recurs as the outcome indicator and selects three commonly used machine learning algorithms: logistic regression, random forest, and XGBoost to construct prediction models. By comprehensively comparing and analyzing the performance of each model based on their unique characteristics and advantages, optimal results are obtained to improve the accuracy and practicality of the models. The results of this study show that the random forest model performs best in terms of various evaluation indicators in both the modeling and validation groups, suggesting that random forest is the optimal model. Studies by Vu and Xiao also indicate that the random forest model has good prediction capabilities when dealing with binary classification problems [31, 32]. Random forest reduces the risk of overfitting by constructing multiple decision trees and introducing randomness. Although XGBoost also has mechanisms to resist overfitting, it may still be difficult to completely avoid overfitting in cases with smaller sample sizes. Additionally, compared to traditional regression models, random forest can better capture nonlinear relationships and complex interactions between variables. In this study, the prediction model based on random forest not only demonstrated good prediction capabilities in the modeling group but also exhibited high sensitivity and specificity in the validation group, indicating that the model has high clinical application value in predicting the risk of TN recurrence after PBC.

While the constructed model has demonstrated promising predictive performance, its translation into a clinical tool—such as a visual web page or miniprogram—remains a goal for future development. Due to ongoing validation and the need for further refinement, the tool has not yet been fully deployed. Future efforts will focus on transforming the model into a practical, user‐friendly application to help clinicians screen high‐risk individuals in a timely manner and facilitate early intervention.

However, several limitations of this study should be noted. First, the retrospective, single‐center design may introduce recall bias in data collection and analysis, potentially compromising the generalizability of the findings. This issue is compounded by the limited patient diversity inherent in a single‐institution study. Second, the relatively small and geographically concentrated sample may not adequately represent broader or more heterogeneous populations. Variations in surgical techniques among clinicians could also introduce additional bias.

Although the model shows initially satisfactory predictive performance, certain constraints remain. One key limitation is the short follow‐up period of only 4–5 months, which may not fully reflect the typical long‐term recurrence pattern of TN—often observed 1.5–2 years after the procedure. As a result, many patients may still be in the “honeymoon phase” following PBC, during which recurrence is less likely to be detected. Future studies should consider extending the follow‐up duration to more accurately assess long‐term recurrence rates and the effectiveness of PBC over time. Another limitation lies in model interpretability. Although the random forest model achieved higher accuracy, we opted for a logistic regression‐based nomogram due to its simplicity and greater clinical applicability. While techniques such as SHAP (Shapley Additive exPlanations) could improve the interpretability of machine learning models such as random forests, they were not employed in this study. Future research could incorporate SHAP or similar methods to enhance model transparency and clinical utility.

Given these limitations, we strongly recommend that future studies adopt a multicenter, prospective design to validate the model’s predictive performance across diverse patient populations and clinical settings. Extending the follow‐up duration will also be essential to more accurately assess long‐term recurrence rates and the sustained effectiveness of PBC. Such efforts would strengthen the model’s external validity and broaden its applicability.

## 5. Conclusion

The results of this study indicate that disease duration, pain classification, balloon shape, compression duration, and delayed disappearance of pain are influencing factors for TN recurrence after PBC, while facial numbness is a protective factor against TN recurrence after PBC. Based on these factors, three classic machine learning algorithms were selected to construct and validate prediction models, among which the random forest model demonstrated the best predictive performance. It can better screen out high‐risk individuals for TN recurrence after PBC, providing support for early identification and intervention.

## Ethics Statement

This retrospective study was approved by the ethics committees of the Fourth Affiliated Hospital of Harbin Medical University and the First Affiliated Hospital of Harbin Medical University (2023‐YXLLSC‐23). All patient data were de‐identified to ensure privacy, and informed consent was obtained from all patients prior to data collection. Patients were informed about the purpose of data collection and their voluntary participation, with the right to withdraw at any time without consequences. The study complied with the Declaration of Helsinki and relevant ethical guidelines to protect patient rights and confidentiality.

## Disclosure

All authors finalized the manuscript, refined the article, and contributed to the final manuscript and have approved it for publication.

## Conflicts of Interest

The authors declare no conflicts of interest.

## Author Contributions

Ying Guo: conceptualization, methodology, funding acquisition, and writing–original draft preparation. Jing Feng: data curation and writing–original draft preparation. Yige Ma: visualization and investigation. Na Zhang: supervision. Jianheng GU: software and validation. Zhaoting Pei: formal analysis.

## Funding

The present study was funded by the Construction and Application of a Quality Sensitivity Index System for Nursing Care in Interventional Treatment of Trigeminal Neuralgia Supported by the Natural Science Foundation of Heilongjiang Province (Grant No. LH2022H006).

## Data Availability

The data that support the findings of this study are available on request from the corresponding author. The data are not publicly available due to privacy or ethical restrictions.
